# Development of New Biosorbent Based on Crosslinked Chitosan Beads with High Brilliant Blue FCF Removal Efficiency

**DOI:** 10.3390/molecules30020292

**Published:** 2025-01-13

**Authors:** Fatiha Lahgui, Beatriz Delgado Cano, Antonio Avalos Ramirez, Michèle Heitz, Hafida Hadjar, Samia Kaddour

**Affiliations:** 1 LSMTM, Laboratoire de Synthèse Macromoléculaire et Thio-organique Macromoléculaire, Faculty of Chemistry, University of Sciences and Technology Houari Boumediene USTHB, Algiers 16111, Algeria; lahf2901@usherbrooke.ca (F.L.); mimakaddour@gmail.com (S.K.); 2 Department of Chemical and Biotechnological Engineering, Faculty of Engineering, Université de Sherbrooke, Sherbrooke, QC J1K 2R1, Canada; michele.heitz@usherbrooke.ca; 3 CNETE, Centre National en Électrochimie et en Technologies Environnementales, Shawinigan, QC G9N 6V8, Canada; beatriz.delgado12@gmail.com; 4 CRAPC, Centre de Recherche Scientifique et Technique en Analyses Physico-Chimiques, Tipaza 42004, Algeria; hafida.hadjar75@gmail.com; 5 LPCMAE, Laboratoire d’Etude Physico-Chimique des Matériaux et Application à l’Environnement, Faculty of Chemistry, University of Sciences and Technology Houari Boumediene USTHB, Algiers 16111, Algeria

**Keywords:** biosorption, chitosan, crosslinking, epichlorohydrin, brilliant blue FCF

## Abstract

Effluents containing synthetic anionic dyes can pose a risk to ecosystems, and they must be treated before their release to the environment. Biosorption, a simple and effective process, may be a promising solution for treating these effluents. In this work, chitosan beads were crosslinked with epichlorohydrin to produce a highly stable and performant biosorbent to remove Brilliant Blue FCF dye. The biosorbent was characterized by determining the functional groups on its surface, as well as its elemental composition, crystallinity, and surface morphology. Crosslinking with epichlorohydrin significantly improved the biosorption capacity of chitosan beads. A maximum biosorption capacity of 600 mg/g corresponding to 99% removal efficiency was observed at pH 3.0, a biosorbent dose of 0.5 g/L, an initial dye concentration of 300 mg/L, a contact time of 10 h, and a temperature of 323 K. The biosorption of Brilliant Blue FCF dye in chitosan beads crosslinked with epichlorohydrin was well described by the Langmuir isotherm and followed an adsorption kinetic of pseudo second order. The thermodynamic parameters indicate a spontaneous biosorption process. The presence of anions such as NO_3_^−^ and SO_4_^2−^ could interfere with the biosorption of Brilliant Blue FCF on the chitosan crosslinked beads, but Cl^−^ did not interfere in biosorption process. Over three biosorption/desorption cycles, the biosorbent showed a removal efficiency of 97% and a desorption rate of over 98%. Chitosan is available worldwide and is a low-cost biomaterial, presenting high potential to be used as a biosorbent to treat industrial effluents containing anionic compounds, such as dyes.

## 1. Introduction

The industrial production of food, textiles, care products, cosmetics, etc., requires the use of dyes to improve their appearance and incite their consumption. Nowadays, the global production of dyes is higher than 7 million tons per year, with over 10,000 varieties of them produced. Approximately 1 to 2% (wt.) of dyes are lost during their synthesis, and 10 to 15% (wt.) are lost during their application, where they are discharged in effluents [1]. From an environmental perspective, effluents charged with dyes represent a risk to aquatic and plant life due to their high stability and resistance to degradation [2]. In fact, several dyes exceed toxic thresholds for organisms and their ecosystems and for human beings. Moreover, the presence of dyes in waterways is highly visible even at trace levels (<1 mg/L), leading to aesthetic pollution and reducing light penetration, which adversely affects photosynthesis [3,4,5,6]. Brilliant Blue FCF (BBr FCF) is a synthetic colorant derived from petrochemicals, and it is widely used as a food colorant for sweets, drinks, dairy products, etc. BBr FCF dye belongs to the triphenylmethane family, the oldest class of synthetic dyes, and it has been chosen as a reference compound for scientific studies of dye emissions and environmental fate. It should be noted that little attention has been given to removing this class of dyes from liquid effluents, as they have a complex structure that increases their resistance to chemical and biological treatments.

The removal of dyes from effluents is a technological challenge. Several processes have been developed, for example, coagulation–flocculation, advanced oxidation, membrane filtration, electrochemical reduction, and exchange resins [7,8,9,10,11]. However, for dye removal, these processes are expensive, complex, and can generate other residues [12,13]. Among these processes, adsorption is a promising option thanks to its simplicity and high efficiency. Currently, liquid–solid biosorption has been the subject of considerable research looking for a potential method for the removal of toxic pollutants from aqueous solutions. The process involves the direct transfer of mass from a liquid phase to a solid phase, a mechanism that is similar to that of traditional adsorption processes using adsorbents such as activated carbon, alumina, silica, or zeolites [14]. However, in biosorption, the material used as an adsorbent is of biological origin, which are inexpensive and widely available in nature [15]. They have complexation and chelation properties and a high adsorption capacity [15].

Chitosan (Cs) is a biomaterial with interesting characteristics for producing solid biosorbents, such as its high adsorption capacity, chemical stability, biodegradability, worldwide availability, and economic feasibility [16]. In addition, it presents a cationic nature in acidic media, being the only cationic polymer that naturally occurs. This explains its high capacity to retain different compounds, such as heavy metals [17,18,19,20], ionic dyes [21,22], phosphate [19,20], and perchlorate [23].

The low mechanical resistance and high solubility of Cs in diluted acid solutions limit the use of Cs as a biosorbent for controlling dyes in effluents, because this kind of effluent mainly exists in the form of acid streams [14]. To overcome this, Cs-based biosorbents can be modified to increase their mechanical and chemical resistance. Kaur K and Jindal R (2019) used nanocomposite-based graphene oxide nanosheets incorporated in a matrix of carboxy-methylcellulose and chitosan without crosslinking for the adsorption of cationic dyes; this technique permitted the use of the biosorbent without affecting its stability [24]. Another technique can be used: it is possible to create a three-dimensional network via covalent bonding between functional groups of Cs and a chemical agent. This modification is called crosslinking. For example, Huang G.H et al. (2019) used genipin to reticulate composite-based copper sulfide nanoparticles/chitosan nanofiber, which improved the stability of composites over a wide pH range and allowed for the removal of tetracycline by catalytic degradation [25]. The use of genipin in the crosslinking of Cs is beneficial because it is a natural, biodegradable, and non-toxic crosslinking agent [26]. Zhou Z et al. (2014) prepared magnetic nanoparticles of chitosan crosslinked with glutaraldehyde to eliminate FD&C Blue 1 (Brilliant Blue FCF) and D&C Yellow 5 from aqueous solutions [27]. Crosslinking agents such as genipin and glutaraldehyde react with -NH_2_ groups of Cs. However, -NH_2_ groups of Cs participate in the adsorption of anionic compounds, and the crosslinking decreases the number of -NH_2_ groups available for capturing anionic dyes such as BBr FCF [28]. For this reason, it is necessary to carry out the crosslinking using an agent that reacts also with the hydroxyl groups (-OH) of Cs, such as epichlorohydrin (ECH) [29]. In the literature, it is widely reported that ECH is highly reactive with Cs hydroxyl groups [15,30]; this is an advantage because ECH does not eliminate the amine groups that are the binding sites for pollutant molecules [14]. Yu X et al. (2021) developed biosorbent-based beads of chitosan hydrogel crosslinked with epichlorohydrin for perchlorate removal from aqueous solution. The authors reported that this method preserves the NH_2_ groups of Cs, which are the main adsorption sites for anionic compounds such as perchlorate and BBr FCF [23].

The main objective of this research is to study the effect of two different concentrations of added ECH crosslinking agent on the properties of Cs and its adsorption capacity towards BBr FCF dye. The best ECH concentration was then selected for further analyses of its improved properties, as well as parametric studies such as the effect of the pH of the solution, biosorbent dose, contact time, temperature, and thermodynamic conditions. Mathematical kinetics and isotherm models were also applied. It is important to note that the biosorption of BBr FCF on Cs beads crosslinked with ECH has not yet been reported in other scientific studies. Chitosan-based biosorbents crosslinked with ECH have significant adsorption potential for anionic pollutants. These biosorbents are characterized by their chemical stability and the presence of amine groups on their surface, which mainly contribute to the specific adsorption of negatively charged pollutants such as anionic dyes. In addition to increasing the mechanical and chemical resistance of Cs, the crosslinking improved the adsorption capacity and led to the production of adsorbents that can be easily regenerated for use in several adsorption–desorption cycles.

## 2. Results

### 2.1. Effect of Epichlorhydrin Concentration on Adsorption Capacity of Chitosan

**Figure 1:**
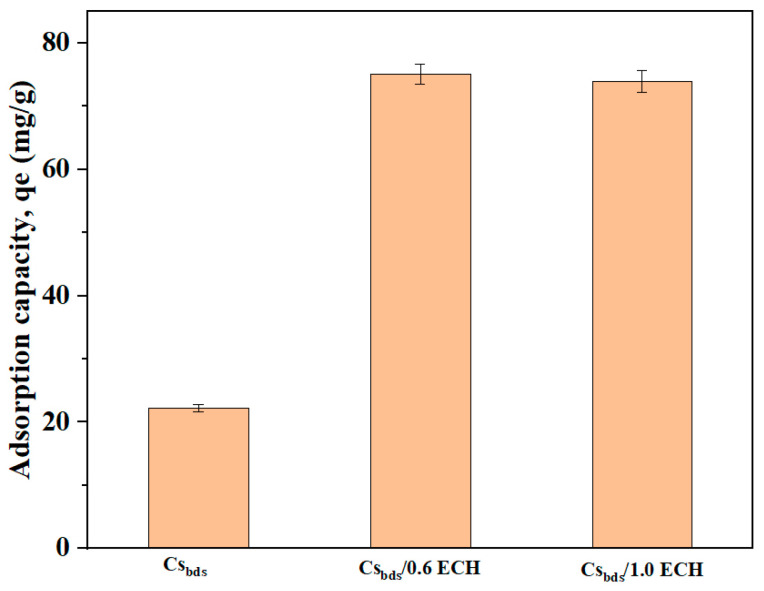
Effect of ECH concentration of crosslinking Cs_bds_ on adsorption capacity at pH 5.5, initial concentration of BBr FCF of 100 mg/L, biosorbent dose of 1 g/L, contact time of 24 h, and room temperature.

The effect of crosslinking Cs_bds_ with epichlorohydrin (ECH) on the adsorption of BBr FCF was studied using ECH at concentrations of 0.6 and 1% *v*/*v*. The adsorption capacity of crosslinked Cs_bds_ for both concentrations of ECH is shown in Figure 1; the non-crosslinked Cs_bds_ is also shown to compare as a blank.

The qe for Cs_bds_ was 22 mg/g, and it increased up to 75 mg/g for Cs_bds_/0.6 ECH and 74 mg/g for Cs_bds_/1.0 ECH. The functional groups on the surface of unmodified Cs_bds_ beads are not accessible to the retained dye molecules. This behavior is probably due to the highly crystalline structure of Cs, as the hydroxyl (-OH) and amine (-NH_2_) groups form inter- and intramolecular hydrogen bonds, leading to an ordered structure [14]. The crosslinking improved the adsorption capacity of modified Cs_bds_ because this reaction consists of creating bonds between the ECH and -OH and/or -NH_2_ groups of the Cs, reducing the hydrogen bonds between the Cs_bds_ chains. This leads to the exposure of unreacted groups, making them more available for the retention of pollutants [29]. This behavior was observed by Yu, X. et al. (2020), the adsorption of perchlorate on chitosan hydrogel beads crosslinked with ECH was higher than on non-crosslinked hydrogel beads [23]. The present study shows that the increase in ECH concentration from 0.6 to 1.0% *v*/*v* did not influence the adsorption capacity, which was nearly the same for both ECH concentrations at around 75 mg/g. In order to minimize the amount of ECH used in the preparation of modified biosorbent, the ECH concentration of 0.6% *v*/*v* was selected for further study. It will be referred to as Cs_bds_/ECH.

### 2.2. Characterization of Cs Beads

#### 2.2.1. Functional Groups

**Figure 2:**
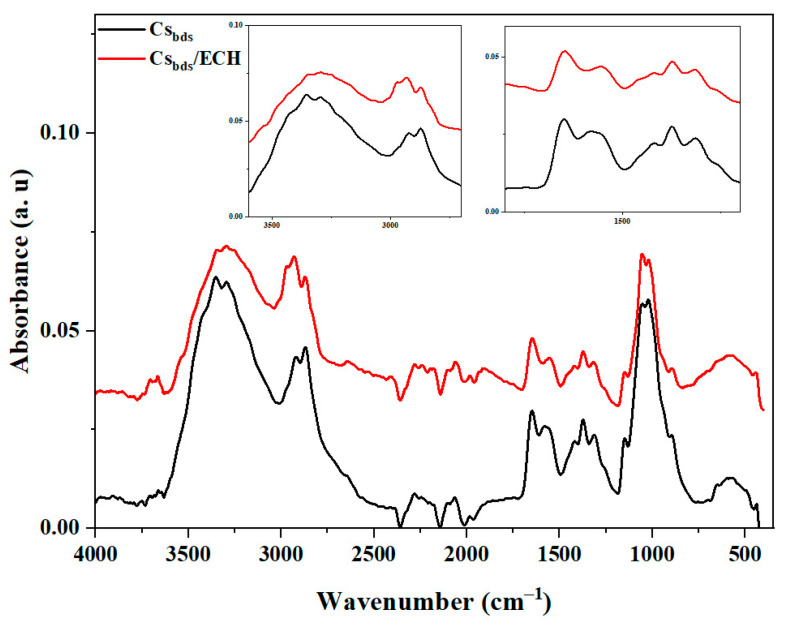
FTIR spectra of the Cs_bds_ and Cs_bds_/ECH biosorbents.

The functional groups of the Cs_bds_ and Cs_bds_/ECH biosorbents were studied by Fourier-transform infrared spectroscopy (FTIR). The spectra are shown in Figure 2.

The FTIR spectrum of Cs_bds_ presents a strong band in the 3289–3344 cm^−1^ region (elongation of NH_2_ and OH groups and intra- and intermolecular hydrogen bonds), peaks at around 2932 and 2865 cm^−1^ (symmetrical and asymmetrical C-H elongation, respectively), bands at around 1649 cm^−1^ (C=O stretching of amide I) and 1315 cm^−1^ (C-N stretching of amide III) corresponding to N-acetyl groups, a band at 1581 cm^−1^ (groups N-H) corresponding to free primary amine, bands at 1423 and 1373 cm^−1^ (CH_2_ elongation and C-O stretching, respectively) corresponding to primary alcohol, a band at 1150 cm^−1^ (asymmetric stretching of the C-O-C bridge) associated with polysaccharides, and bands at 1050 and 1020 cm^−1^ (C-O stretching). All these peaks have been observed for Cs in other studies [31,32,33].

The spectrum of Cs_bds_/ECH presented all the above-mentioned peaks, with some differences in the level of intensity. The most important of these differences are the decreases in the intensity of the band in the 3289–3344 cm^−1^ region, the peak at 1581 cm^−1^, and the peaks at 1423 and 1373 cm^−1^, corresponding to the NH_2_ and OH groups, free primary amine NH, and primary alcohol CH_2_OH, respectively. A weak peak at 1100 cm^−1^ also appeared in the spectrum of Cs_bds_/ECH corresponding to the vibration of C-H of ECH. The slight decrease in the intensity of peaks characteristic of Cs_bds_/ECH compared with Cs_bds_ can be attributed to the reaction of certain OH and/or NH_2_ groups with ECH, so the crosslinked biosorbent has less free OH and/or NH_2_ groups [34].

#### 2.2.2. Elemental Composition

The content of carbon, hydrogen, and nitrogen (C, H and N) in Cs_bds_ and Cs_bds_/ECH was determined by elemental analysis. The values of C, H, and N content are given in Table 1.

After crosslinking, the carbon content decreased from 41.05 to 40.92% (wt.), while the nitrogen content decreased from 7.41 to 7.07% (wt.). In contrast, the hydrogen content increased from 8.01 to 8.41% (wt.). The changes in carbon, hydrogen, and nitrogen content are probably attributed to the reaction of certain Cs functional groups with ECH during crosslinking. But these changes were minor, confirming that the crosslinking reaction of Cs_bds_ by ECH did not affect the density of the functional groups on the Cs_bds_/ECH surface [28], especially that of the NH_2_ groups, which are responsible for the retention of the dye molecules. 

#### 2.2.3. Crystallinity of Cs Beads

**Figure 3:**
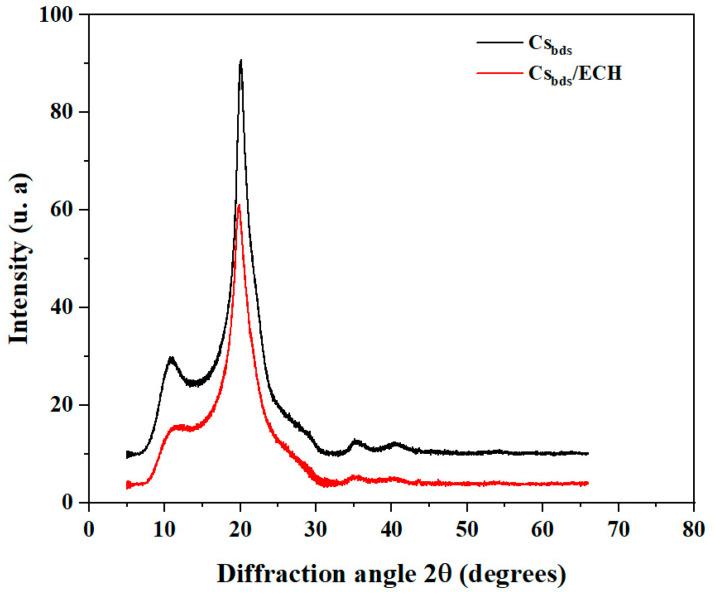
XRD diffractograms of Cs_bds_ and Cs_bds_/ECH.

The amorphous and semi-crystalline structures of Cs_bds_ and Cs_bds_/ECH were analyzed by diffractometry of X-ray (XRD). The XRD diffractograms are shown in Figure 3 in the 2Ɵ range of 5° to 65°. The X-ray diffractograms of Cs_bds_ and Cs_bds_/ECH show two characteristic peaks at 2Ɵ 10° and 20°, confirming the presence of semi-crystalline structures in Cs_bds_ and Cs_bds_/ECH. The biosorbents were characterized by high crystallinity, which is probably due to the ordered structure of the polysaccharide, where hydroxyl and amino groups can form strong inter- and intramolecular hydrogen bonds [35]. But for Cs_bds_/ECH, the peaks at 2Ɵ 10° and 20° were less intense than for Cs_bds_.; this decrease in crystallinity can be explained by the introduction of ECH, because the crosslinking of Cs by ECH lead to the formation of bridges between the Cs chains by the generation of bonds between the functional groups of Cs chains and ECH. This creates a certain disorder in the structure of Cs chains. As such, their crystallinity decreased, as reported by Crini G et al. (2019) [14].

#### 2.2.4. Surface Morphology

**Figure 4:**
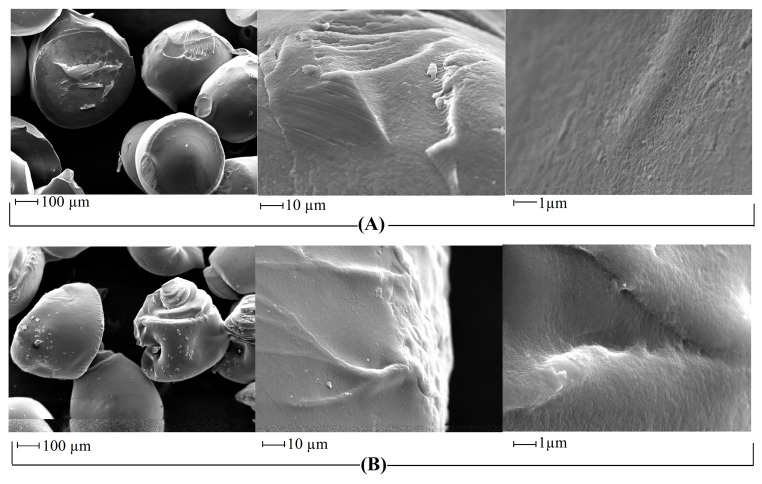
SEM micrographs of (**A**) Cs_bds_ and (**B**) Cs_bds_/ECH.

The surface morphology of Cs_bds_ and Cs_bds_/ECH was analyzed by scanning electron microscope. Figure 4 shows the surface of both kinds of beads at three levels of magnification. 

Figure 4 shows that the beads of Cs_bds_ and Cs_bds_/ECH were not perfectly spherical. Both kinds of beads were relatively smooth and presented an ovoidal form. In the case of Cs_bds_/ECH, the bead surface presented folds and waves in one extreme, forming more-irregular particles than Cs_bds_. These observations are in accord with the literature, as research has indicated that Cs crosslinked with ECH produces rough and heterogeneous particle surfaces [36].

#### 2.2.5. Stability in Acid Solutions

The stability of Cs_bds_ and Cs_bds_/ECH in acid solutions was analyzed using acetic acid (CH_3_COOH), hydrochloric acid (HCl), nitric acid (HNO_3_), and sulfuric acid (H_2_SO_4_) solutions at concentrations of 0.5, 1.0, and 2.0 M, and deionized water.

The Cs_bds_ beads were soluble in all concentrations of CH_3_COOH solutions, as well as in HCl and HNO_3_ solutions at 0.5 M. However, they were insoluble in HCl and HNO_3_ solutions at 1.0 and 2.0 M; H_2_SO_4_ solutions at 0.5, 1.0, and 2.0 M; and deionized water. The Cs_bds_/ECH beads were insoluble in all the acid solutions and in deionized water.

These results confirm that the crosslinking of Cs by ECH limited its dissolution in acidic solutions. During the crosslinking reaction, certain OH and/or NH_2_ groups of the Cs_bds_/ECH are blocked by the ECH forming bridges between the biosorbent chains, thus creating a three-dimensional network in all three directions, which reduces the mobility of chain segments. The material then becomes insoluble in an acid solution [37].

#### 2.2.6. The Point of Zero Charge (pH_pzc_)

**Figure 5:**
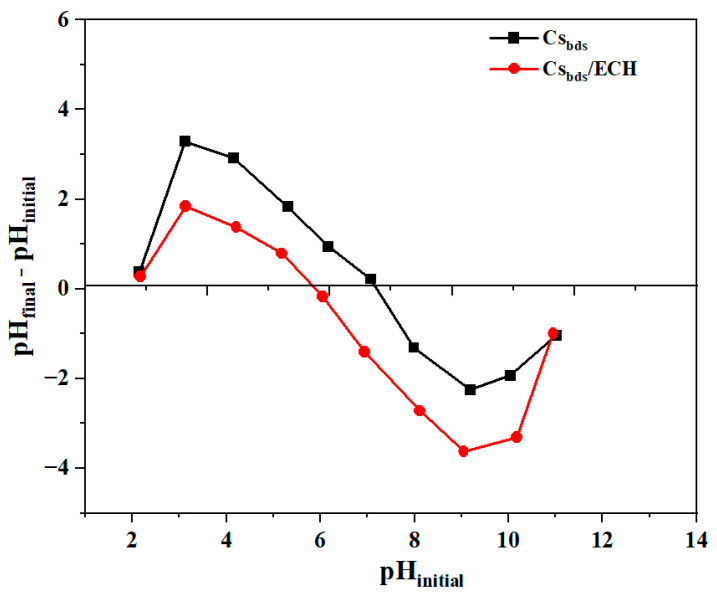
Isoelectric pH (pH_pzc_) of Cs_bds_ and Cs_bds_/ECH.

The isoelectric pH (pH_pzc_) of biosorbents was determined by plotting pH_f_-pH_i_ as a function of pH_i_. Figure 5 shows the pH_pzc_ of both Cs_bds_ and Cs_bds_/ECH. The pH_pzc_ corresponds to the condition where the net surface charge of the biosorbent is zero [38]. This means that the pH of the media is not changed by the functional groups presented on the surface of the biosorbent by the capture or release of cations and anions, such as H^+^ or OH^−^. The isoelectric pH is important because it indicates the pH range where the biosorbent will present the best adsorption capacity for charged compounds.

The pH_pzc_ of Cs_bds_ and Cs_bds_/ECH were 7 and 6, respectively. This means that crosslinking with ECH might result in an increase in the negative charge on the surface, which could contribute to the electrophilicity of ECH. In another study, the pH_pzc_ of ECH-crosslinked chitosan hydrogel beads was 5.1 [23]. At pH levels below the pH_pzc_, the biosorbent surface was covered by protons (H^+^), causing the protonation of -NH_2_ amino groups according to the following equation: -NH_2_ + H^+^ = -NH_3_^+^. As such, the surface of the biosorbent became positively charged. At pH levels higher than the pH_pzc_, pH_f_ was lower than pH_i_, and the Cs_bds_ and Cs_bds_/ECH biosorbent surface was covered by hydroxide groups (OH^−^); this means that the biosorbent surface became negatively charged [39]. For this reason, BBr FCF (an anionic dye) will be better adsorbed at pH levels below the pH_pzc_, where electrostatic interactions will occur between the positive -NH_3_^+^ groups of the biosorbent and the negative sulfonic groups (-SO_3_^−^) of BBr FCF.

#### 2.2.7. Degree of Swelling

The swelling degree (S) of Cs_bds_ and Cs_bds_/ECH submerged in water was determined. It was 53 and 34% (wt.) for Cs_bds_ and Cs_bds_/ECH, respectively. The swelling degree of Cs_bds_/ECH was inferior to that of Cs_bds_. The swelling corresponds to the mass of liquid adsorbed by the adsorbent matrix. In the case of Cs, the interactions of hydrophilic groups such as -OH and -NH_2_ groups with water caused their retention. As crosslinking slightly reduces the density of these groups on the surface of Cs_bds_/ECH, its degree of swelling was lower [23].

### 2.3. BBr FCF Adsorption on Cs_bds_/ECH

#### 2.3.1. pH of Media

**Figure 6:**
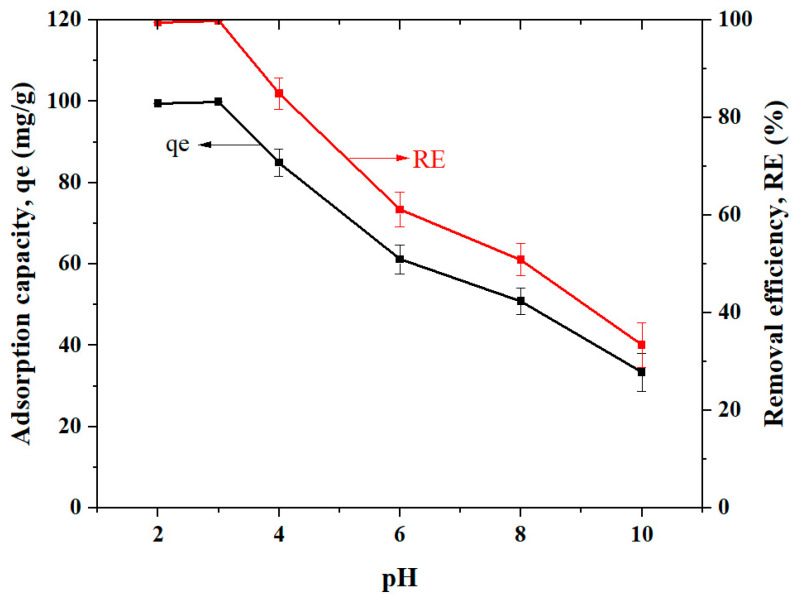
Effect of pH on BBr FCF removal as pH ranged from 2.0 to 10.0, at initial concentration of BBr FCF of 100 mg/L, biosorbent dose of 1.0 g/L, contact time of 24 h, and room temperature.

The effect of pH on the adsorption capacity of Cs_bds_/ECH is shown in Figure 6. The maximum qe of 100 mg/g was observed at pH 3.0, which corresponded to 99.5% RE of BBr FCF on Cs_bds_/ECH. The qe decreased with pH from 100 to 33 mg/g as pH was adjusted from 3.0 to 10.0, respectively.

The adsorption capacity of BBr FCF on Cs_bds_/ECH was higher at acidic than at basic pH. This agrees with the previous discussion regarding pH_pzc_, which indicated that when pH < pH_pzc_, the surface of the biosorbent was positively charged and attracted the negative sulfonic groups of BBr FCF via an electrostatic interaction process. According to Figure 5, the highest positive charge corresponded to pH 3.0, as well as the highest qe, as shown in Figure 6.

The mechanism of biosorption of BBr FCF on Cs_bds_/ECH is represented in Equations (1)–(3) as follows [40]:

(a) Protonation of amin groups of Cs_bds_/ECH at pH 3
  (1) $$R—{NH}_{2}+H^{+}⇄R—{NH}_{3}^{+}$$
(b) Dissociation of BBr FCF in aqueous solution
  (2) $$D—{SO}_{3}Na+H_{2}O⇄D—{SO}_{3}^{-}+{Na}^{+}$$
(c) Electrostatic interaction (in red) between NH_3_^+^ and SO_3_^−^
  (3) R—NH3++D—SO3−⇄R—NH3+ˊˊˊˊˊˊˊˊˊˊˊˊO3−S—D

According to results of this section, the pH of 3.0 was selected to perform the next assays.

#### 2.3.2. Dose of Cs_bds_/ECH

**Figure 7:**
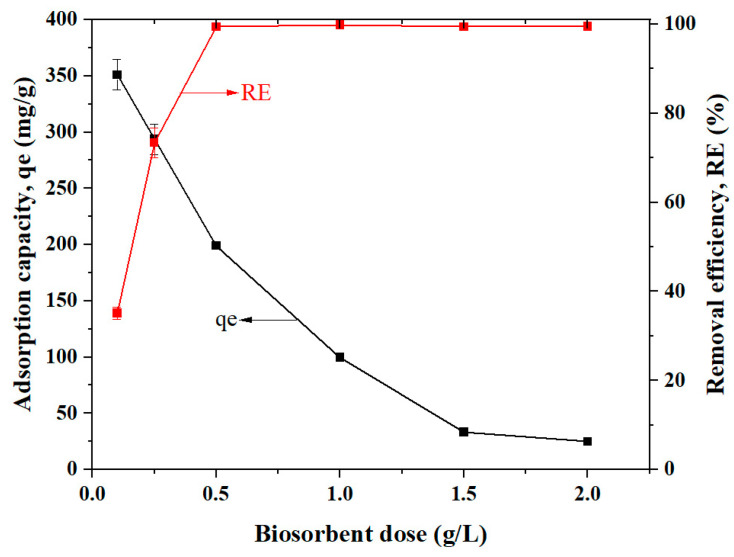
Effect of biosorbent dose on BBr FCF adsorption, with the dose ranging from 0.1 to 2.0 g/L, at pH 3.0, BBr FCF initial concentration of 100 mg/L, contact time of 24 h, and room temperature.

The effect of the Cs_bds_/ECH dose on the BBr FCF adsorption was studied by varying the biosorbent dose from 0.1 to 2.0 g/L. The adsorption capacity at equilibrium for each dose and the removal efficiency as a function of Cs_bds_/ECH dose are shown in Figure 7. The qe of BBr FCF on Cs_bds_/ECH decreased from 350.96 mg/g for a dose of 0.1 g/L to 50 mg/g for 2.0 g/L. Meanwhile, the RE of BBr FCF on Cs_bds_/ECH increased from 36 to 99% when the dose increased from 0.1 to 0.5 g/L and was nearly stable in the dose range of 0.5 to 2.0 g/L, with a value close to 99%. The increase in RE when the dose ranged from 0.1 to 0.5 g/L was probably due to the availability of more adsorption sites to retain BBr FCF molecules [32]. At a dose of 0.5 g/L, the removal efficiency of BBr FCF on Cs_bds_/ECH was over 99%, indicating that at this dose, the biosorbent possessed enough adsorption sites to capture all the dye molecules in the solution. Increasing the dose of adsorbent can lead to its aggregation when it is in the form of particles, and therefore, the RE of pollutants decreases [32]. In this study, the biosorbent was in the form of beads and did not present aggregates, and for this reason, the RE was not affected. In the case of qe, it is calculated by dividing the mass of the solute (mg) by the mass of biosorbent added (g). As mentioned before, for the lowest dose of biosorbent tested, there were enough active sites to remove nearly 100% of the dye. Thus, the increase in biosorbent dose will not increase the removal of the dye. Increasing the dose caused a lower value of qe to be obtained because the dose increased for a fixed volume with a fixed mass of adsorbate without ever saturating the biosorbent [41]. According to these results, the dose of 0.5 g/L was selected to carry out the equilibrium studies.

#### 2.3.3. Initial Concentration of BBr FCF

**Figure 8:**
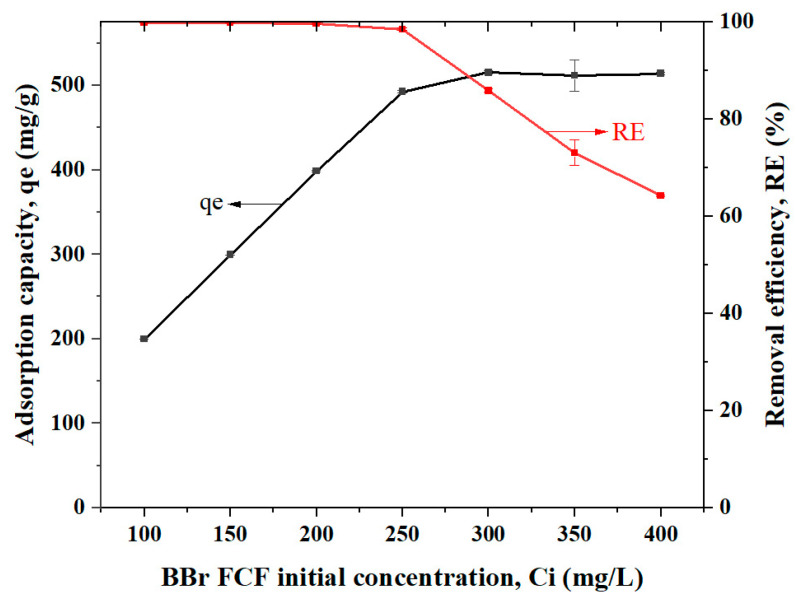
Effect of initial concentration (Ci) on BBr FCF adsorption when Ci ranged from 100 to 400 mg/L, at pH 3.0, biosorbent dose of 0.5 g/L, contact time of 24 h, and room temperature.

The effect of initial concentration (Ci) of BBr FCF on the adsorption capacity of Cs_bds_/ECH was studied by varying Ci from 100 to 400 mg/L (Figure 8). The adsorption capacity increased with Ci from 199 to 515 mg/g when the Ci ranged from 100 to 300 mg/L. Then, the adsorption capacity was nearly stable when Ci ranged from 300 to 400 mg/L. On the contrary, the RE of BBr FCF on Cs_bds_/ECH was around 100% when Ci ranged from 100 to 250 mg/g, while it progressively decreased to 64% when Ci ranged from 250 to 400 mg/L.

The qe increased linearly when Ci ranged from 100 to 250 mg/L because the biosorbent had enough adsorption sites to capture BBr FCF molecules without being saturated. In the moment that the biosorbent was saturated, the adsorption capacity stayed nearly constant because the biosorbent could not take more adsorbate, and a dynamic equilibrium was established. This was confirmed by the evolution of the RE: at concentrations lower than 250 mg/L, the available adsorption sites could capture all the molecules of BBr FCF, presenting a value around 100%. However, an increase in Ci to over 250 mg/L caused the saturation of adsorption sites and the removal efficiency to decrease linearly.

#### 2.3.4. Contact Time and Temperature

**Figure 9:**
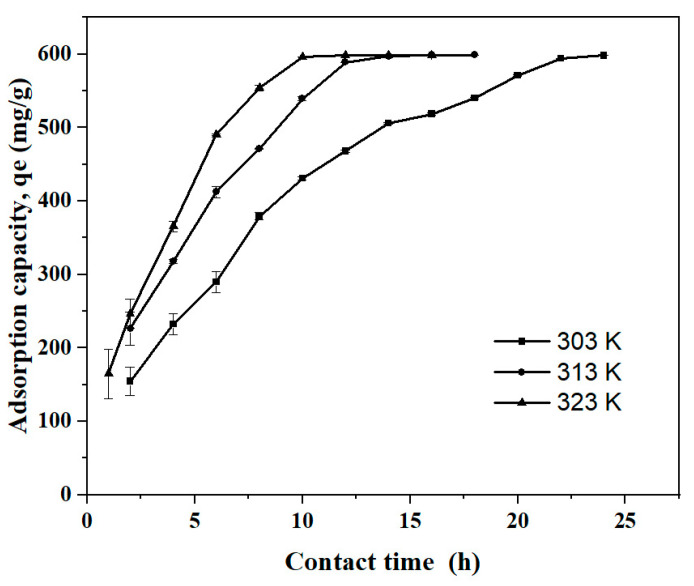
Effect of temperature and contact time on adsorption equilibrium at a pH of 3.0, initial BBr FCF concentration of 300 mg/L, and biosorbent dose of 0.5 g/L.

Figure 9 shows the effect of temperature and contact time on the adsorption capacity of Cs_bds_/ECH at equilibrium. The adsorption capacity increased with time until the BBr FCF was completely adsorbed and a dynamic equilibrium was reached. The contact time required to reach the equilibrium of BBr FCF adsorption on Cs_bds_/ECH was 22 h at 303 K, 14 h at 313 K, and 10 h at 323 K. The increase in temperature accelerated the rate of BBr FCF adsorption and consequently reduced the contact time required to reach equilibrium. This can be explained by the fact that temperature increases the energy of BBr FCF molecules, causing higher diffusion through the biosorbent and accelerating contact between BBr FCF molecules and the adsorption sites [42].

The maximum adsorption capacity was similar for the three temperatures tested at equilibrium: the qe was around 600 mg/g, corresponding to a RE of over 99%. On an industrial scale, the adsorption process is preferably carried out at ambient temperature in order to decrease the cost of the process [30]. In contrast, the increase in temperature from 303 to 323 K decreased by half the time needed to completely remove the BBr FCF, which can allow for the use of smaller processing equipment and consequently lower cost.

### 2.4. Adsorption Isotherms

**Figure 10:**
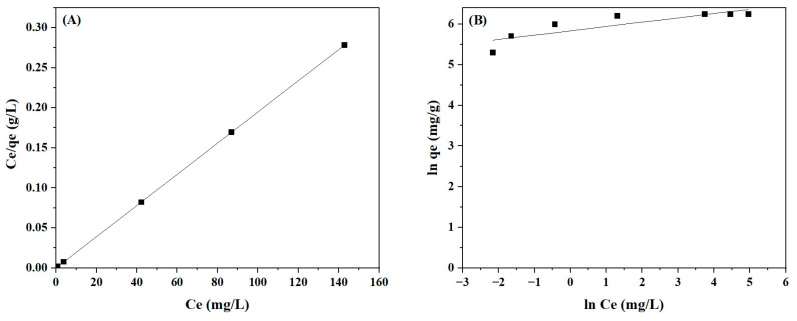
Representation of isotherm adsorption by (**A**) Langmuir model and (**B**) Freundlich model.

The linear fitting of Langmuir and Freundlich isotherms for the adsorption of BBr FCF on Cs_bds_/ECH is shown in Figure 10, and their values are shown in Table 2.

The fitting of these isotherm models shows that the adsorption isotherms are well described by the Langmuir isotherm, presenting for this model a coefficient of correlation close to unity. The maximum adsorption capacity calculated with this model was 555 mg/g, which was close to the experimental value of 515 mg/g. The Langmuir separation factor R_L_ parameter was calculated for the entire initial dye concentration range of 100 to 400 mg/L. If R_L_ values are in the range 0 < R_L_ < 1, they indicate a favorable adsorption; if R_L_ > 1, the adsorption is unfavorable; R_L_ = 1 indicates a linear adsorption, and RL = 0 corresponds to an irreversible adsorption [43]. The values in this study were in the range of 0 < R_L_ < 1, indicating a favorable adsorption of BBr FCF on Cs_bds_/ECH. The Langmuir model corresponds to a monolayer adsorption process on a homogeneous adsorbent surface, with energetically equal adsorption sites and no interaction between adsorbed molecules [44].

### 2.5. Adsorption Kinetics

**Figure 11:**
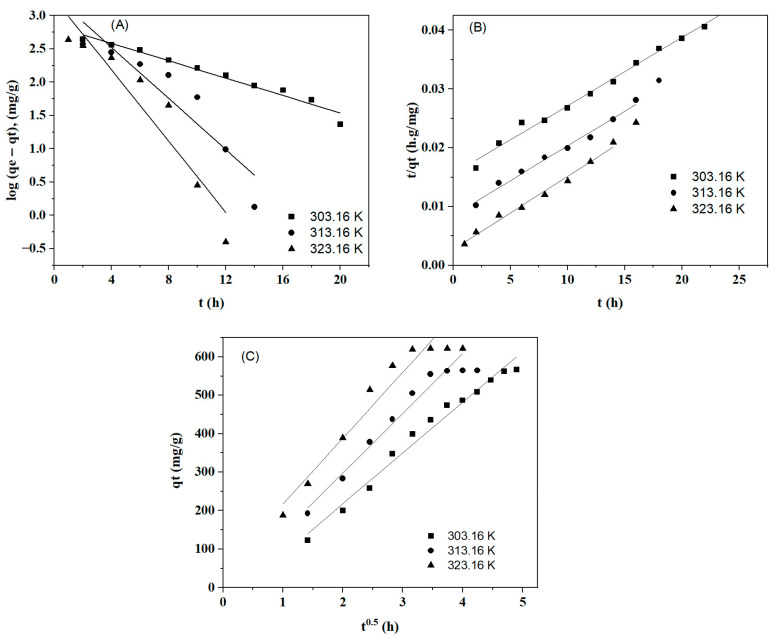
Linear fitting of adsorption kinetic models: (**A**) pseudo-first-order, (**B**) pseudo-second-order, and (**C**) intraparticle diffusion.

The kinetic models of pseudo first order, pseudo second order, and intraparticle diffusion were applied to studying the adsorption of BBr FCF on Cs_bds_/ECH. The values of the parameters for each model are shown in Table 3. The linear fitting of the pseudo-first-order, pseudo-second-order, and intraparticle diffusion kinetic models are presented in Figure 11.

The kinetics of BBr FCF adsorption on Cs_bds_/ECH when temperature ranged from 303 to 323 K was better represented by the PSO than the PFO model; the PSO adjusted better, with a coefficient of determination R^2^ of 0.99 for all temperatures. In the case of PFO, the R^2^ varied from 0.84 to 0.97, adapting better at 303 K. The adsorption capacity calculated with both models was higher than the experimental value of 600 mg/g, ranging from 708 to 1813 mg/g for PFO and from 769 to 862 mg/g for PSO. This shows again that PSO produced values closer to the experimental values than PFO. The better applicability of the PSO model may indicate that the adsorption of BBr FCF on Cs_bds_/ECH is a chemical process. The rate constants k_1_ and k_2_ of the PFO and PSO models increased slightly with temperature, confirming that the adsorption rate of BBr FCF increased with temperature [45].

The adsorption of BBr FCF on Cs_bds_/ECH can be controlled using the following steps: I. mass transfer from the liquid phase to the outside layer of the biosorbent; II. bulk intraparticle diffusion of BBr FCF molecules into the pores on the Cs_bds_/ECH surface; III. the adsorption process. The third step is generally rapid, so the adsorption process is limited by the first or the second step [46]. The plot of the linearized model of intraparticle diffusion can provide information about the adsorption-limiting step. The plot presents two sections: one with a linear increase and one with a plateau. This suggests that intraparticle diffusion is involved in the adsorption of BBr FCF on Cs_bds_/ECH. Since the plot did not pass through the origin, the intraparticle diffusion was not the only step controlling the adsorption process; this may indicate that the adsorption of BBr FCF on Cs_bds_/ECH is a complex process that is limited both by the mass transfer and intraparticle diffusion [47]. In addition, the rate constant K_3_ increased with temperature, confirming again that BBr FCF adsorption rate increases with temperature.

**Figure 12:**
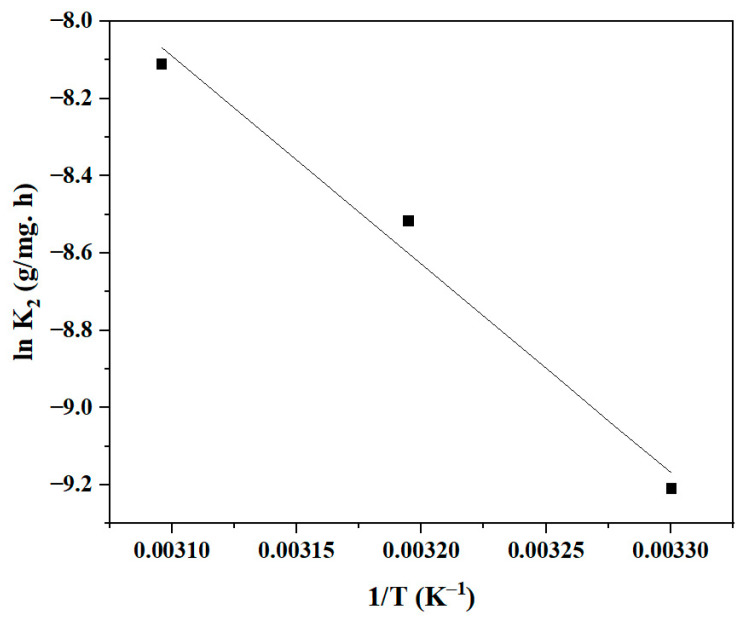
Graphical representation of linear Arrhenius equation for BBr FCF biosorption on Cs_bds_/ECH.

The calculation of the PSO rate constant K_2_ at different temperatures and the Arrhenius equation can be used to calculate the activation energy E_a_ of the BBr FCF biosorption process on Cs_bds_/ECH [48]. The relationship between K_2_ and E_a_ is given in Equation (4).

ln K_2_ = −(E_a_/RT) + ln A (4)

where R is the universal gas constant (8.314 J/mol. K), T (K) is the absolute temperature, and A is the Arrhenius constant. The plot of K_2_ as a function of 1/T is shown in Figure 12. The plot is a linear curve with a correlation coefficient of 0.98. E_a_ can be calculated from the slope. The E_a_ of the biosorption of BBr FCF on Cs_bds_/ECH was 43.81 kJ/mol. The results of this study suggest that the interaction between the BBr FCF molecule and the functional groups on the Cs_bds_/ECH surface requires 43.81 kJ/mol of energy, which is the lowest energy required for a specific adsorbate/adsorbent interaction to take place [49].

A value of E_a_ from 8 to 50 kJ/mol can indicate physisorption, while a value between 60 and 800 kJ/mol can indicate chemisorption [50,51]. The biosorption of BBr FCF on Cs_bds_/ECH may be considered a kind of physisorption. In this study, the E_a_ was close to the superior limit of physisorption, which may suggest strong biosorbent/adsorbate interactions, mainly electrostatic interactions. For example, the E_a_ of the adsorption of methylene blue on the hazelnut shell, which involves electrostatic interactions, was found to be 45.6 kJ/mol [50].

### 2.6. Thermodynamic Parameter

**Figure 13:**
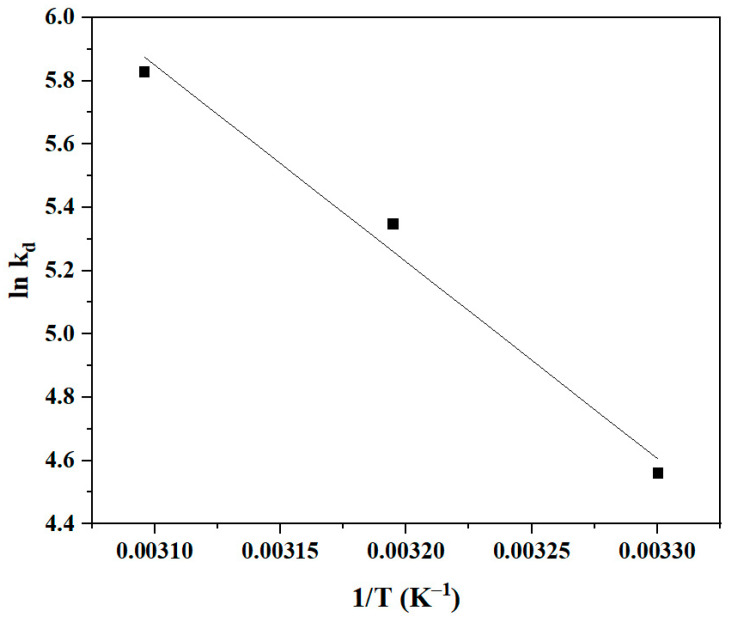
The plot of Van’t Hoff equation for BBr FCF biosorption on Cs_bds_/ECH.

The thermodynamic parameters of BBr FCF adsorption on Cs_bds_/ECH, including the Gibbs free energy change (∆G°), enthalpy change (∆H°), and entropy change (∆S°), were calculated (Table 4) from the plot of the Van’t Hoff equation (ln K_d_ as function of (1/T)), as showed in Figure 13.

The plot of ln K_d_ as a function of (1/T) is a linear function with a correlation coefficient of 0.99. The three parameters are shown in Table 4. The enthalpy change (∆H°) of 51.71 (kJ/mol) and the entropy change (∆S°) of 0.21 (kJ/mol. K) were calculated from the slope and y-intercept, respectively. The standard Gibbs free energy (∆G°) was −11.31, −13.26, and −14.45 (kJ/mol) at 303, 313, and 323 K, respectively. The negative ∆G° indicates a spontaneous adsorption of BBr FCF on Cs_bds_/ECH [52]. The ∆G° decreased (became more negative) with temperature, confirming that BBr FCF was better adsorbed as temperature increased [53,54]. Positive ∆H° values suggest an endothermic adsorption process [55].

### 2.7. Effect of Salts on Adsorption Capacity

The interference effect of dissolved salts (NaCl, NaNO_3_, Na_2_SO_4_) on the biosorption of BBr FCF on Cs_bds_/ECH was studied, and the results are shown in Table 5.

Table 5 shows the effect of dissolved salts (NaCl, NaNO_3_, Na_2_SO_4_) on the biosorption of BBr FCF on Cs_bds_/ECH. The adsorption capacity of BBr FCF on Cs_bds_/ECH was 591 mg/g without salt, whereas it was 590, 562, and 557 mg/g in the presence of NaCl, NaNO_3_, and Na_2_SO_4_, respectively. The presence of dissolved salts in the solution can result in a high ionic strength, which can exert a substantial influence on the entire adsorption process. This influence is stronger when adsorption is achieved by electrostatic interactions [56,57]. At an acidic pH, the interactions between the negatively charged BBr FCF molecules and the positively charged NH_2_ groups of Cs_bds_/ECH are electrostatic interactions. The presence of ions such as NO_3_^−^ and SO_4_^2−^ in the solution can weaken the electrostatic adsorbate/adsorbent interactions, as these ions can be in competition with the adsorbate molecules to occupy the adsorption sites. As a result of this interference, the qe of BBr FCF on Cs_bds_/ECH decreased. In the case of Cl^−^, no decrease in qe was observed. Wang, H et al. (2021) reported that SO_4_^2−^ ions are characterized by high hydrability and therefore high polarizability; NO_3_^−^ ions have medium hydrability, resulting in medium polarizability; while Cl^−^ ions have low hydrability and polarizability. In other terms, the hydrability and polarizability of the anions increases their adsorption potential on the surface of the positively charged biosorbent and therefore their interference with the biosorption of BBr FCF [58].

### 2.8. Biosorbent Regeneration

**Figure 14:**
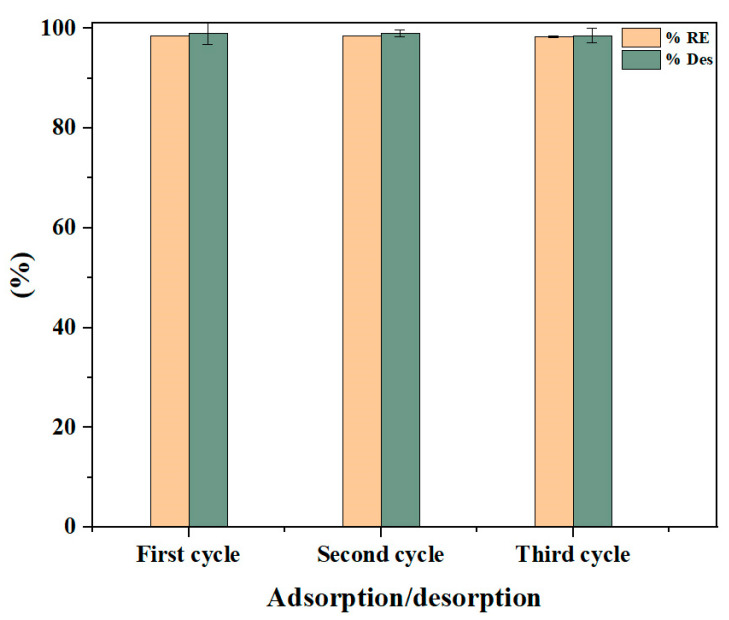
Biosorption/desorption of BBr FCF on Cs_bds_/ECH; adsorption: pH 3.0, initial BBr FCF concentration of 300 mg/L, biosorbent dose of 0.5 g/L, contact time of 48 h, and room temperature; desorption: NaOH concentration of 0.5 M, contact time of 48 h, and room temperature.

In order to investigate the potential of regenerating the Cs_bds_/ECH, three biosorption/desorption cycles were carried out. The results are shown in Figure 14. In the three biosorption/desorption cycles, the BBr FCF removal efficiency of Cs_bds_/ECH was over 97%, while the desorption rate was around 98%. In the three cycles, the BBr FCF molecules were completely detached from the Cs_bds_/ECH adsorption sites. This was due to the fact that in the alkaline solution, the NH_3_^+^ groups of Cs_bds_/ECH were deprotonated, resulting in a weakening of the electrostatic adsorbate/biosorbent interactions; consequently, the BBr FCF molecules were desorbed [27]. The desorption mechanism is represented in Equation (5) as follows:

(5) R—NH3+ˊˊˊˊˊˊˊˊˊˊˊˊO3S—D−+NaOH⇄R—NH2+D—SO3Na

The Cs_bds_/ECH reserved its BBr FCF removal efficiency after desorption in alkaline solution during the three biosorption/desorption cycles. This was probably due to the release of all the adsorption sites on the surface of the biosorbent, which became available for the retention of BBr FCF molecules after desorption. These results confirm that Cs_bds_/ECH can be regenerated without affecting its BBr FCF removal efficiency, which is an important economic parameter as it can reduce the cost of the adsorption process.

### 2.9. Interaction Between BBr FCF and Cs_bds_/ECH

**Figure 15:**
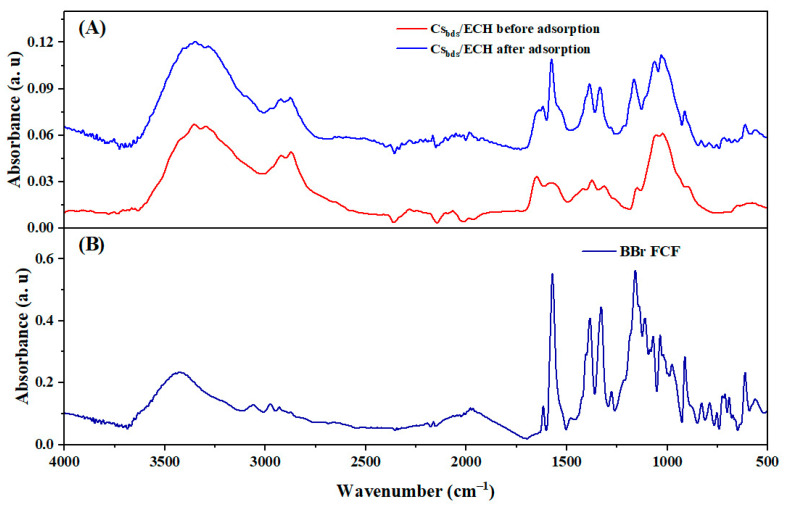
FTIR spectrum of (**A**) Cs_bds_/ECH before and after adsorption of BBr FCF and (**B**) BBr FCF dye.

The Cs_bds_/ECH beads charged with the BBr FCF dye were characterized by FTIR in order to study the modifications produced in functional groups of Cs_bds_/ECH after adsorption. The spectra of the Cs_bds_/ECH before and after adsorption are presented in Figure 15. The spectrum of BBr FCF is also shown.

The spectrum of Cs_bds_/ECH changes after BBr FCF adsorption (Figure 15A), especially in the wavenumber range of 1600 to 500 cm^−1^. In this region, some peaks appeared after adsorption, specifically at 1620 cm^−1^ (elongation of -C=C-), 1571 cm^−1^ (elongation of -CN), 1160 cm^−1^ (S=O elongation), 723 cm^−1^ (=CH deformation), and 609 cm^−1^ (SO_3_^−^ groups). All these peaks are present in the BBr FCF spectrum (Figure 15B). At pH 3, the BBr FCF molecules are retained on the Cs_bds_/ECH surface through electrostatic interactions between the -NH_3_^+^ groups of the biosorbent and the -SO_3_^−^ groups of the dye. This retention of dye on the biosorbent surface was confirmed by the appearance of new peaks corresponding to BBr FCF in the Cs_bds_/ECH spectrum after biosorption.

### 2.10. Comparison of BBr FCF Adsorption on Other Biosorbents

The biosorption of BBr FCF has been studied using other materials, such as biomaterials (biosorbent based on chitosan and bentonite), agricultural residues (bottom ash obtained during the combustion of the coal, de-oiled soya cake, hen feathers, walnut shell powder obtained during combustion, hazelnut skin, and a composite of polyaniline/hazelnut skin), and activated carbon from forestry residual biomass (Table 6). In all these studies, BBr FCF adsorption was performed at acidic pH levels ranging from 2.0 to 3.0, confirming that adsorption is predominated by electrostatic interactions between the positively charged biosorbent surface and the sulfur groups of BBr FCF, which become negatively charged in solution (-SO_3_^−^). The adsorption capacity of these biosorbents varied across a wide range from 0.5 to 1600 mg/g. The chitosan-based biosorbents showed a higher BBr FCF adsorption capacity than most of the biosorbents reported thanks to the presence of NH_2_ groups on their surface, which are protonated at pH values ranging from 2 to 3 and interact strongly with the anionic groups of BBr FCF. The adsorption capacity of the Cs_bds_/ECH developed in this work presented a high adsorption capacity of up to 600 mg/g. The results of this study confirm that the Cs_bds_/ECH developed is an effective biosorbent for the removal of anionic dyes from aqueous solutions.

## 3. Discussions

The adsorption of BBr FCF using a new biosorbent based on Cs was studied. The new biosorbent was produced via a crosslinking reaction of Cs beads using ECH as the crosslinking reagent. The study of the functional groups by FTIR and the elemental composition of Cs_bds_/ECH by elemental analysis showed that the crosslinking reaction with ECH did not change the basic structure of Cs_bds_ but was sufficient to limit its solubility in acid solutions, via the creation of bridges between its chains, and to improve its adsorption capacity, by exposing its functional groups, which had not reacted during crosslinking. The biosorption of BBr FCF on Cs_bds_/ECH was optimized by following a series of parametric studies, obtaining an experimental maximum adsorption capacity of 600 mg/g corresponding to a removal efficiency of 99%. The adsorption of BBr FCF on Cs_bds_/ECH was produced predominantly by electrostatic interactions between the protonated -NH_2_ groups of the biosorbent and the sulfonic groups of BBr FCF. The biosorption of BBr FCF on Cs_bds_/ECH was well described by the Langmuir isotherm and followed adsorption kinetics of pseudo second order. According to the thermodynamic parameters, the biosorption of BBr FCF on Cs_bds_/ECH is spontaneous. The presence of Cl^−^ anions did not interfere with the biosorption of BBr FCF on Cs_bds_/ECH, but the presence of NO_3_^−^ and SO_4_^2−^ can slightly decrease the adsorption capacity of Cs_bds_/ECH for BBr FCF. The biosorbent developed can be regenerated without reducing or losing its BBr FCF adsorption capacity. These results confirm that crosslinking Cs with ECH produces a high-performance biosorbent that can be regenerated and reused to remove ionic compounds from aqueous solutions, such as the anionic dye.

## 4. Materials and Methods

### 4.1. Biosorbent and Reagents

**Figure 16:**
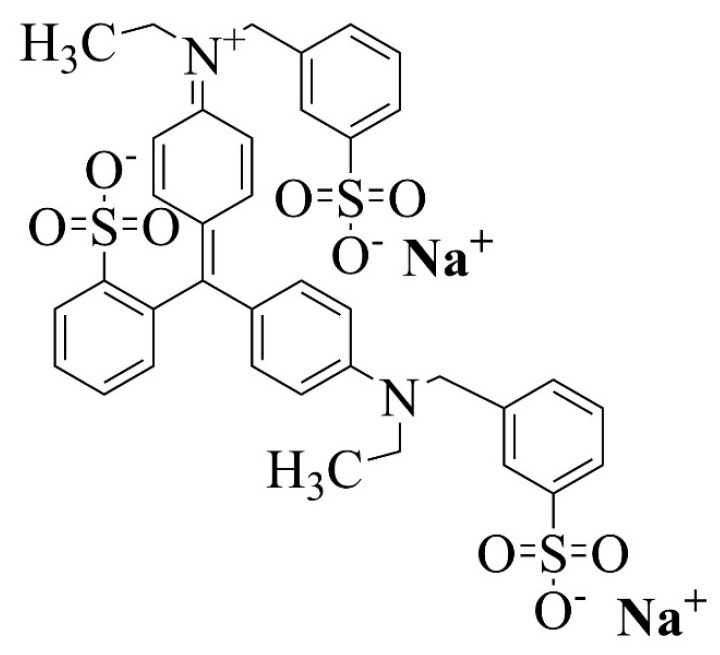
The 2D chemical structure of disodium bis(4-(ethyl[(3-sulfonatophenyl)methyl]amino)phenyl)(2-sulfonatophenyl)methylium (BBr FCF) with the molecular formula C_37_H_34_N_2_Na_2_O_9_S_3_.

Chitosan with a molecular weight of 50,000 to 190,000 Da and a deacetylation degree (DD) of 75% was used for all the assays (Sigma-Aldrich, Burlington, MA, USA). Acetic acid (CH_3_COOH, 99% *v*/*v*), sodium hydroxide (NaOH, 50% *v*/*v*), hydrochloric acid (HCl, 37% *v*/*v*), nitric acid (HNO_3_, 71% *v*/*v*), sulfuric acid (H_2_SO_4_, 78% *v*/*v*), sodium chloride (NaCl), sodium nitrate (NaNO_3_), sodium sulfate (Na_2_SO_4_) (all of them from Fisher Scientific, Waltham, MA, USA), and Epichlorohydrin (C_3_H_5_ClO, 99% *v*/*v*) (Sigma-Aldrich, Burlington, MA, USA) were the reagents used to produce the beads and perform the adsorption assays, as defined below. Disodium bis(4-(ethyl[(3-sulfonatophenyl)methyl]amino)phenyl)(2-sulfonatophenyl)methylium food grade (Brilliant Blue FCF, Shree Nath Ji Dyestuffs, New Delhi, India), with the molecular formula C_37_H_34_N_2_Na_2_O_9_S_3_ and the 2D chemical structure shown in Figure 16, was used to produce synthetic polluted effluents. Solutions were prepared using deionized water (Millipore Simplicity™, Fisher Scientific, Waltham, MA, USA).

### 4.2. Chitosan Beads

#### 4.2.1. Preparation of Chitosan Beads

The Cs beads were prepared using the method adapted by Rorrer GL et al. (1993) [68] and by Guibal E et al. (1998) [69]. Briefly, 2 g of Cs were placed in 100 mL of CH_3_COOH solution 2% *v*/*v* and stirred until complete dissolution was achieved. The Cs solution was dropped into 200 mL of NaOH solution 2 M using a MasterFlex L/S peristaltic pump (Cole-Parmer, Vernon Hills, IL, USA). The Cs beads were formed when the drops of Cs solution came in contact with the NaOH solution. The Cs beads stayed in the NaOH solution for 24 h; then, they were washed several times with deionized water until the pH of the washing water was around 7.0. The pH was determined with an AB250 model pH meter (Fisher Scientific, Waltham, MA, USA). Chitosan beads were filtered and dried overnight in an oven at 30 °C. Chitosan beads without modifying by crosslinking were identified as Cs_bds_.

#### 4.2.2. Preparation of Chitosan Beads Crosslinked with Epichlorohydrin (ECH)

**Figure 17:**
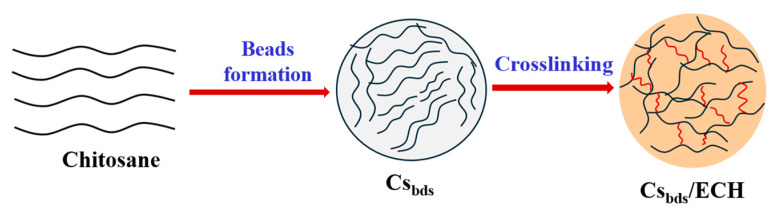
Schematical representation of formation of Cs beads and Cs crosslinking beads.

**Figure 18:**
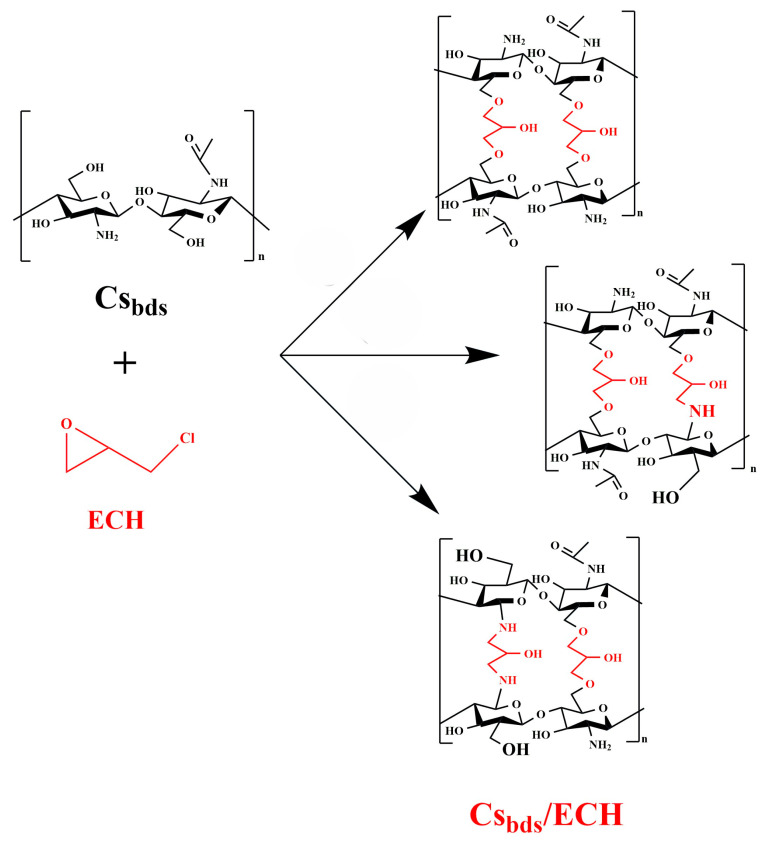
Reactions between the Cs beads and ECH [14,32].

The Cs_bds_ were crosslinked as follows [70]: 10 g of wet Cs_bds_ was added to 100 mL of ECH solution. The concentrations of ECH tested were 0.6 and 1.0% *v*/*v*. The pH of the ECH solution was adjusted to 10.0 using a solution of NaOH 0.1 M. The duration of the crosslinking reaction was 2 h, with an agitation of 100 rpm and a temperature of 40 °C. The crosslinked beads were washed several times with deionized water to eliminate the residual ECH; they were filtered and dried overnight at 30 °C in an oven. The crosslinked beads were identified as Cs_bds_/0.6 ECH and Cs_bds_/1.0 ECH, according to the concentration of ECH used. The Cs bead formation is schematically shown in Figure 17 and the possible reactions between the Cs beads and ECH are illustrated in Figure 18. 

### 4.3. Effect of ECH Concentration on BBr FCF Adsorption Capacity

Biosorption tests of BBr FCF on Cs_bds_, Cs_bds_/0.6 ECH, and Cs_bds_/1.0 ECH were carried out under the following conditions: pH 5.5, an initial BBr FCF concentration of 100 mg/L, an adsorbent dose of 1 g/L, a contact time of 24 h, and room temperature. The aim of these assays was to analyze the effect of ECH concentration during the Cs crosslinking on the BBr FCF adsorption capacity. These biosorption tests allowed us to select the best ECH-crosslinked Cs_bds_ that offered the highest adsorption capacity for use in the rest of the study.

### 4.4. Methods for Characterizing Cs Beads

The Cs_bds_ and Cs_bds_/ECH were characterized to determine their main morphological and physicochemical parameters. The Cs_bds_ was characterized for comparison with modified beads.

The functional groups of Cs_bds_ and Cs_bds_/ECH were identified by Fourier-transform infrared (FTIR) spectroscopy analysis in the 500–4000 cm^−1^ wavenumber range using a Nicolet iS5 spectrometer with an iD7 ATR diamond accessory and OMNIC Software (Thermo Fisher scientific, Waltham, MA, USA). The content of carbon, hydrogen, and nitrogen (C, H, and N) in Cs_bds_ and Cs_bds_/ECH was determined by elemental analysis employing a 2400 Series II CHNS/O analyzer (Perkin Elmer, Waltham, MA, USA). The surface morphology of Cs_bds_ and Cs_bds_/ECH was analyzed by scanning electron microscopic analysis (SEM) employing an EVO MA10 microscope (Zeiss, Oberkochen, Germany). The images were taken at a working distance of 9 mm with an electron high tension of 20 kV. Before analyses, the samples were coated with gold to improve their conductivity. The crystallinity was determined by X-ray diffraction (XRD) using a D2 Phaser diffractometer (Bruker, Bremen, Germany) equipped with DIFFRAC.Eva V5.1 software. Patterns were recorded from 5° to 65° with a scan speed of 0.3°/s, using a CuKα monochromatic radiation source.

Chitosan is soluble in diluted solutions of organic acids and certain mineral acids. To analyze the effect of the crosslinking reaction on the stability of beads in diluted acid solutions, qualitative analyses were carried out by adding Cs_bds_ and Cs_bds_/ECH to solutions of CH_3_COOH, HCl, H_2_SO_4_, and HNO_3_ at concentrations of 0.5, 1.0, and 2.0 M, using deionized water as a blank. A mass of 0.5 g of Cs_bds_ and Cs_bds_/ECH was added to 50 mL of each acid solution and deionized water. The suspensions were stirred at room temperature for 24 h.

The point of zero charge (pH_pzc_) of Cs_bds_ and Cs_bds_/ECH was determined using the salt addition method [71,72]. Briefly, a mass of 0.1 g of each biosorbent was added to 50 mL of NaCl 0.01 M solution. The initial pH was adjusted from 2 to 11 using solutions of H_2_SO_4_ 0.1 M or NaOH 0.1 M. The suspensions were stirred for 24 h, and the final pH (pH_f_) was determined. The pH_pzc_ was calculated by plotting pH_f_ − pH_i_ versus pH_i_, where the intersection of pH_f_ − pH_i_ with the X-axis corresponded to the pH_pzc_.

To analyze the biosorbents’ affinity to water, the degree of swelling of Cs_bds_ and Csbds/ECH in deionized water was determined. Briefly, 0.5 g of biosorbent was added to 50 mL of deionized water and stirred for 24 h at room temperature. The biosorbent was filtered using filter paper and weighed. The degree of swelling was calculated according to Equation (6):

S = 100 × (m_s_ − m_d_)/m_s_ (6)

where S is swelling (% wt.) and m_s_ (g) and m_d_ (g) are the mass of swollen and dry biosorbent beads, respectively.

### 4.5. Adsorption of BBr FCF on Selected ECH-Crosslinked Cs

#### 4.5.1. Parametric Studies

Firstly, 2 L of a stock solution of Brilliant Blue FCF at a concentration of 2000 mg/L was prepared in deionized water. This solution was used to prepare dye solutions at different concentrations by dilution.

BBr FCF adsorption on Cs_bds_/ECH was performed in a batch system. All tests were carried out in duplicate in Erlenmeyer flasks of 250 mL with magnetic stirring using a WH 260 hot plate/stirrer (Wiggnes, Wuppertal, Germany).

- Effect of pH:

The effect of pH on the BBr FCF adsorption capacity of Cs_bds_/ECH was studied by varying the pH of solutions from 2.0 to 10.0 using 0.1 or 0.01 M of HCl and NaOH solutions.

Parameter values: biosorbent dose = 1 g/L, volume of BBr FCF solution V = 50 mL, initial dye concentration Ci = 100 mg/L, stirring speed = 300 rpm, contact time = 24 h, and room temperature.

- Effect of biosorbent dose:

The impact of the biosorbent dose on BBr FCF adsorption was determined by varying the dose of Cs_bds_/ECH from 0.1 to 2 g/L.

Parameter values: optimum pH, V = 50 mL, Ci = 100 mg/L, stirring speed = 300 rpm, contact time = 24 h, and room temperature.

- Effect of initial BBr FCF concentration:

The effect of the initial dye concentration was studied using concentrations ranging from 50 to 400 mg/L. The data obtained in this study were adjusted using adsorption isotherms.

Parameter values: optimum pH and dose, V = 50 mL, stirring speed = 300 rpm, contact time 24 h, and room temperature.

- Effect of temperature on contact time:

The effect of the temperature on the contact time required to reach equilibrium in the adsorption of BBr FCF was studied from 0 to 24 h at three temperatures of 303, 313, and 323 K under the predetermined optimum conditions. The experimental data obtained in this study were adjusted using adsorption kinetic models. The thermodynamic parameters of the adsorption process of the BBr FCF dye on Cs_bds_/ECH were also determined. These parametric studies are summarized in Table 7.

In each experiment, the remaining dye concentration was determined by UV–visible spectrophotometry at 629 nm using a GENESYS 50 spectrophotometer (Thermo Fisher scientific, Waltham, MA, USA) and quartz cuvettes. A volume was taken from the supernatant and diluted in water before measuring its adsorbance UV–visible. 

The adsorption capacity, qe (mg/g), of biosorbents was calculated using Equation (7):

qe = (Ci − Ce) × V/m(7)

and the BBr FCF removal efficiency, RE (%), using Equation (8):

% RE = 100 × (Ci − Ce)/Ci(8)

where Ci and Ce (mg/L) are the initial concentration and the concentration at equilibrium of BBr FCF. V (L) is the volume of BBr FCF solution. m (g) is the mass of biosorbent.

#### 4.5.2. Adsorption Isotherms

The experimental data obtained during the study of the effect of the initial concentration of BBr FCF on Cs_bds_/ECH were used to adjust the adsorption isotherms and determine the parameters of the Langmuir and Freundlich models. 

The Langmuir isotherm is based on the fact that the adsorbent surface is homogeneous, with energetically uniform binding sites. It assumes that adsorption occurs in monolayers with no interaction between adsorbed molecules [73,74]. The Freundlich isotherm describes multilayer adsorption on heterogeneous surfaces. It assumes that adsorption sites are not energetically equal, with interactions occurring between attached molecules [75,76]. The mathematical expressions of the linear form of the Langmuir and Freundlich isotherms are given in Equations (9) and (10), respectively.

Ce/qe = (1/qm) × Ce + 1/(K_L_ × qm) (9)

lnqe = lnK_F_ + 1/n × lnCe (10)

where Ce (mg/L) is the concentration of BBr FCF at equilibrium. qe and qm (mg/g) are the adsorption capacity of BBr FCF on Cs_bds_/ECH at equilibrium and calculated with the Langmuir isotherm, respectively. K_L_ (L/mg) is the Langmuir adsorption coefficient. K_F_ is the Freundlich constant, indicating the adsorption capacity of the adsorbent. 1/n is a constant representing the affinity of the solute for the adsorbent.

The Langmuir isotherm can be used to determine the feasibility of adsorption by calculating the separation factor (R_L_) [77] according to Equation (11).

R_L_ = 1/(1 + KL × Ci) (11)

where Ci (mg/L) is the initial concentration of BBr FCF and K_L_ (L/mg) is the Langmuir adsorption coefficient.

#### 4.5.3. Adsorption Kinetics

In this study, the kinetic models of pseudo first order, pseudo second order, and intraparticle diffusion were adjusted to experimental data. The pseudo-first-order model (PFO) [Largergren’s model] [78] assumes that adsorption is a physical process [79], while the pseudo-second-order model (PSO) indicates that adsorption is a chemical process [80]. The plot of the linear equation of the intraparticle diffusion model can provide information about the rate-limiting step in adsorption. If the curve passes through the origin, the intraparticle diffusion is the only adsorption-rate-limiting step [81]. The linear equations of pseudo first order, pseudo second order, and intraparticle diffusion are given in Equations (12)—(14), respectively.

log(qe − qt) = log qe − (K_1_/2.303) × t (12)

t/qt = 1/(K_2_ × (qe)^2^) + t/qe (13)

qt = K_3_ × t^^0.5^ + C (14)

where qt and qe (mg/g) are the adsorption capacity of BBr FCF on Cs_bds_/ECH at time (t) and at equilibrium, respectively. K_1_ (h^−1^), K_2_ (g/mg h), and K_3_ (mg/g h^0.5^) are the rate constant of adsorption of pseudo first order, pseudo second order, and intraparticle diffusion, respectively. C is a constant regarding the thickness of the boundary layer.

#### 4.5.4. Thermodynamic Parameters

The adsorption thermodynamic parameters of BBr FCF on Cs_bds_/ECH can provide information about the feasibility and spontaneity of adsorption. The standard Gibbs free energy change (ΔG°), enthalpy change (ΔH°), and entropy change (ΔS°) were determined according to the following equations:

∆G° = −RT ln (K_d_) (15)

K_d_ = Ca/Ce (16)

ln K_d_ = ∆S°/R) − (∆H°/R × T) (17)

where R is the universal gas constant (8.314 J/mol. K). T(K) is temperature. K_d_ is the equilibrium constant. Ce (mg/L) is the concentration of BBr FCF at equilibrium. qe (mg/g) is the adsorption capacity at equilibrium.

#### 4.5.5. Effect of Salts on Adsorption Capacity

BBr FCF is used in the food industry, where liquid effluents contain dissolved salts that can interfere with the molecules targeted by adsorption [82]. To evaluate the effect of the presence of these compounds on the BBr FCF adsorption capacity of Cs_bds_/ECH, biosorption tests were carried out in the presence of sodium chloride (NaCl), sodium nitrate (NaNO_3_), and sodium sulfate (Na_2_SO_4_) salts [83,84,85]. An appropriate quantity of salt was added to solutions of BBr FCF at an initial concentration of 300 mg/L, a pH of 3.0, and a biosorbent dose of 0.5 g/L, then stirred at 300 rpm for 48 h. The concentrations of NaCl, NaNO_3_, and Na_2_SO_4_ tested were 300 mg/L.

#### 4.5.6. Biosorbent Regeneration

The possibility of regenerating Cs_bds_/ECH after the adsorption of BBr FCF was investigated. Briefly, BBr FCF adsorption tests were carried out under the optimum biosorption conditions of pH 3.0, biosorbent dose 0.5 g/L, and initial dye concentration 300 mg/L, under agitation at 300 rpm over 48 h at room temperature. At the end of the biosorption tests, the beads charged with BBr FCF were filtered using filter paper and washed with water to remove any dye molecules not fixed in the surface of the biosorbent. Then, the beads were placed in an Erlenmeyer flask containing 50 mL of a 0.5 M NaOH solution under agitation at 300 rpm over 24 h at room temperature. Three biosorption/desorption cycles were performed.

The rate of desorption (% Des) was determined according to Equation (18):

% Des = 100 × Cd/(Ci − Ce)(18)

where Ci, Ce, and Cd (mg/L) are the initial concentration, the concentration at equilibrium, and the desorbed concentration of BBr FCF, respectively.

In these tests, a solution of BBr FCF of 300 mg/L was prepared in NaOH 0.5 M and analyzed by UV–visible spectrophotometer. The desorbed concentration of BBr FCF was determined at 567 nm using a GENESYS 50 model spectrophotometer (Thermo Fisher scientific, USA) and quartz cuvettes.

## Figures and Tables

**Table 1:** C, H, and N content values for Cs_bds_ and Cs_bds_/ECH.

Content (% wt.)	C	H	N
Cs_bds_	41.05	8.01	7.41
Cs_bds_/ECH	40.92	8.41	7.07

**Table 2:** Parameters of Langmuir and Freundlich isotherms.

Langmuir				Freundlich		
qmax (mg/g)	K_L_ (L/mg)	R_L_	R^2^	K_F_	1/n	R^2^
556	1.06	0.009–0.002	0.999	1.13	0.17	0.75

**Table 3:** Parameters of pseudo-first-order, pseudo-second-order and intraparticle diffusion kinetic models.

Temperature (K)	Kinetic Models								
	Pseudo First Order (PFO)			Pseudo Second Order (PSO)			Intraparticle Diffusion		
	qe (mg/g)	K_1_ (h^−1^)	R^2^	qe (mg/g)	K_2_ (g/mg·h)	R^2^	C	K_3_ (mg/g·h^0.5^)	R^2^
303	708	0.15	0.97	862	0.0001	0.99	−14.47	132	0.98
313	1950	0.44	0.86	806	0.0002	0.99	53.67	143	0.97
323	1813	0.62	0.84	769	0.0003	0.99	54.43	155	0.95

**Table 4:** Thermodynamic parameters of BBr FCF adsorption on Cs_bds_/ECH.

Ln K_d_			∆G° (kJ/mol)			∆S° (kJ/mol. K)	∆H° (kJ/mol)
303 K	313 K	323 K	303 K	313 K	323 K		
4.56	5.35	5.83	−11.31	−13.26	−14.45	0.21	51.71

**Table 5:** qe of BBr FCF adsorption on Cs_bds_/ECH alone and in presence of salts at pH 3.0, initial BBr FCF concentration of 300 mg/L, biosorbent dose of 0.5 g/L, salt concentration of 300 mg/L, contact time of 48 h, and room temperature.

	Without Salt	NaCl	NaNO_3_	Na_2_SO_4_
qe (mg/g)	591 ± 0.1	590 ± 1	562 ± 2	557 ± 3

**Table 6:** Biosorption of BBr FCF on several biosorbents.

Biosorbent	qe (mg/g)	Reference
Chitosan cryogel beads	1600	García-González, A et al. (2021) [59]
Chitosan powder	210	Dotto GL and Pinto LDA (2011) [60]
Magnetic nanoparticles of chitosan crosslinked with Glutaraldehyde	476	Zhou Z et al. (2014) [27]
Chitosan powder	164	Gonçalves JO et al. (2014) [61]
Bottom ash	7	Gupta VK et al. (2006) [62] Gupta VK et al. (2006) [62]
De-oiled soya	18	
Hen feathers	238	Mittal A (2006) [63]
Unmodified Bentonite	6	Hernández-Hernández KA et al. (2013) [64] Hernández-Hernández KA et al. (2013) [64]
Iron-Modified Bentonite	14	
Walnut shell powder	51	Mohan CS et Ahmadi Khatoon B (2017) [65]
Hazelnut skin	0.5	Banimahd Keivani M (2018) [66] Banimahd Keivani M (2018) [66]
Composite Polyaniline/hazelnut skin	0.8	
AC from forestry biomass	14.1	Valladares C et al. (2019) [67]
Crosslinked Cs beads with ECH	600	This work

**Table 7:** Study of the effect of operating parameters on BBr FCF adsorption of selected ECH-crosslinked Csbds.

Operating Parameter	Level				
	pH	Dose (g/L)	Ci (mg/L)	t (h)	T (K)
pH	2–10; (Δ = 2)	1	100	24	298
Dose of Cs_bds_/ECH	Best pH	0.1–2; (Δ = 0.5 g)	100	24	298
Initial concentration (Ci) of BBr FCF	Best pH	Best dose	100–400; (Δ = 50 mg/L)	24	298
Contact time and temperature	Best pH	Best dose	Best Ci	0–24; (Δ = 2 h)	303, 313, 323

## Data Availability

On request, the corresponding authors can provide the data presented in this article.
